# Association between weight-adjusted-waist index and urge urinary incontinence: a cross-sectional study from NHANES 2013 to 2018

**DOI:** 10.1038/s41598-024-51216-2

**Published:** 2024-01-04

**Authors:** Haohao Sun, Jingxi Huang, Hao Tang, Bingbing Wei

**Affiliations:** Department of Urology, Affiliated Wuxi People’s Hospital of Nanjing Medical University, Wuxi, 214023 China

**Keywords:** Bladder, Urological manifestations, Weight management

## Abstract

This study aimed to investigate the association between urge urinary incontinence (UUI) and weight-adjusted waist circumference index (WWI), a newly developed measure of obesity. Data from the 2013–2018 National Health and Nutrition Examination Survey (NHANES) were included in the present cross-sectional study. Urge urinary incontinence was identified by self-reported urine leakage before reaching the toilet. Weighted multivariate logistic regression and generalized additive models were used to investigate the connection between WWI and UUI and its nonlinearity. The nonlinear relationship was explored using smoothed curve fitting. Additionally, further analyses were performed on subgroups and interaction tests were conducted. In the study, a total of 14,118 individuals were enrolled, with a UUI prevalence rate of 21.18%. Overall UUI was more prevalent with elevated WWI (OR 1.20, 95% CI 1.13–12.8, *P* < 0.0001), which similar results were observed in weekly (OR 1.32, 95% CI 1.18–1.48, *P* < 0.0001) and daily (OR 1.27, 95% CI 1.06–1.53, *P* = 0.0091) UUI. And this connection remained steady among all subgroups (*P* > 0.05 for all interactions). Smoothed curve fitting showed no nonlinear relationship between WWI and UUI. In addition, a stronger correlation was found between WWI and UUI risk than other obesity indicators such as waist circumference (WC) and body mass index (BMI). Among US adults, weight-adjusted waist circumference index values are positively associated with elevated odds of UUI and show stronger associations than WC and BMI. Further studies are required to elucidate the causal relationship between WWI and UUI.

## Introduction

Urge urinary incontinence (UUI) is defined by a sudden, irresistible urge to urinate followed immediately by involuntary incontinence^1^. In the United States, UUI affects a substantial portion of the population, with estimates ranging from 9.3% to 30.8% in women and 2.6% to 20.9% in men, and the prevalence of this condition dramatically increases with age^1–3^. UUI can be a crippling condition, leading to severe impairments in psychosocial well-being and self-confidence, as well as a decline in social interactions and interpersonal relationships^2^. Consequently, these symptoms significantly impact the quality of life, often necessitating behavioral, medical, or surgical interventions.

Obesity has emerged as a significant global public health issue, with its prevalence showing a substantial increase in recent decades^4^. It is estimated that by 2030, almost half of all adults in the United States will be classified as obese^5^. Studies have displayed that obesity is an important factor in UUI^6–9^. While body mass index (BMI) has traditionally been the go-to measure for identifying obesity, it falls short in distinguishing between lean mass and fat mass^10,11^. Park et al. proposed a novel anthropometric measure called the weight-adjusted-waist index (WWI), emphasizing waist circumference (WC) while minimizing its association with BMI^12,13^. As a result, the weight-adjusted-waist index (WWI) is a more accurate indicator of weight-independent centripetal obesity. Despite its superiority to BMI in predicting obesity, its relationship with urge urinary incontinence (UUI) remains unexplored.

Therefore, the current study examined the relationship between WWI and UUI in the whole adult population of the United States using data from the National Health and Nutrition Examination Survey (NHANES) carried out between 2013 and 2018.

## Materials and methods

### Data source

Cross-sectional data were obtained from the NHANES, a nationwide program conducted by the National Center for Health Statistics (NCHS) to evaluate the state of nutrition and health in the United States. Utilizing an advanced multistage probability methodology, this program generates a sample of non-institutionalized Americans that accurately represents the entire nation. It involves in-home interviews in which participants provide information on their health, socioeconomic status, and other relevant aspects. Additionally, there are mobile examination facilities where physical and laboratory examinations are conducted.

The NCHS's Research Ethics Review Board has approved all techniques utilized in NHANES investigations, and all individuals surveyed have provided written informed consent. All detailed NHANES study designs and data can be accessed by the public at www.cdc.gov/nchs/nhanes/.

### Study population

**Figure 1:**
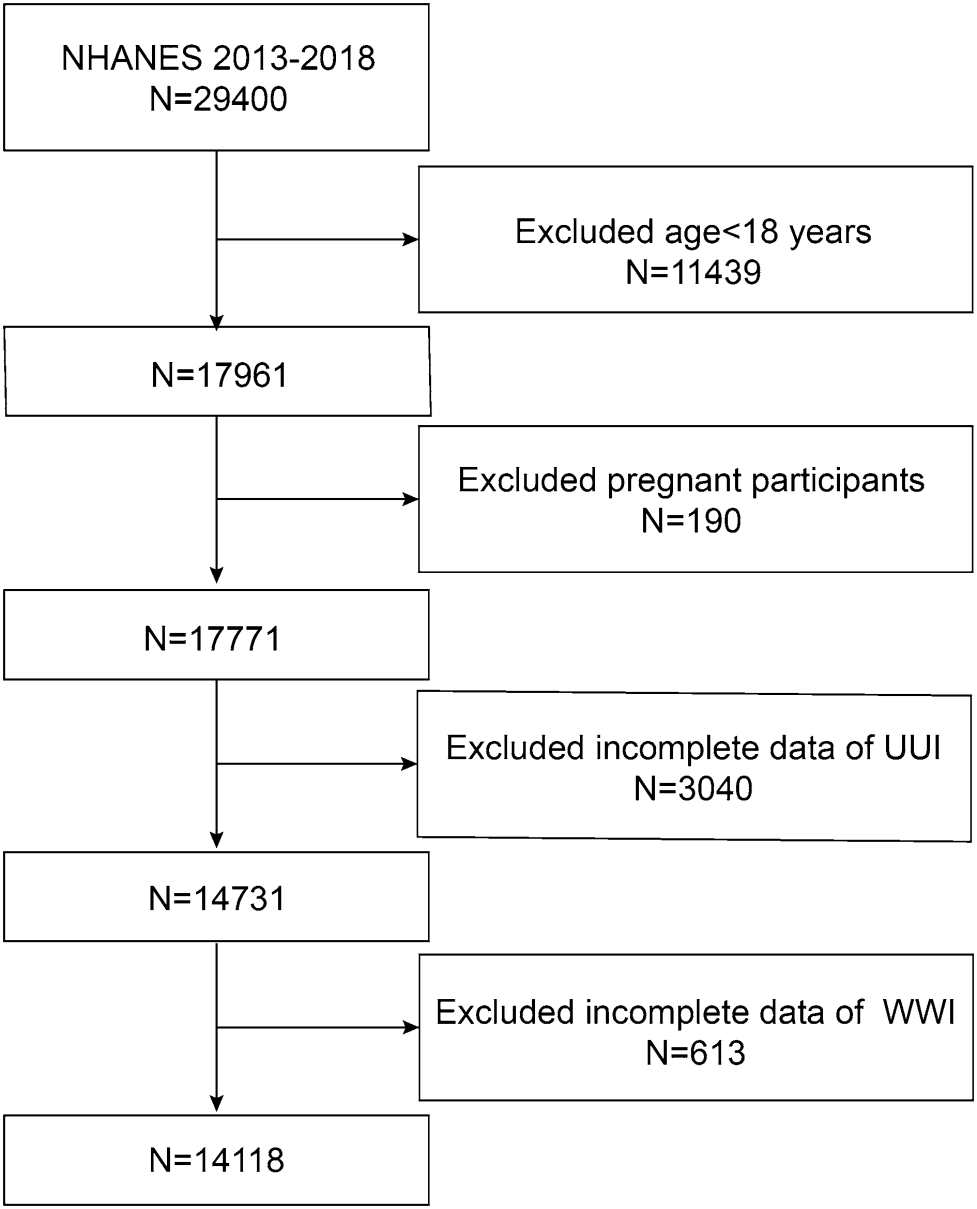
Participant selection criteria. NHANES indicates National Health and Nutrition Examination Survey.

Participants for the research were chosen from NHANES between the years 2013 and 2018. Our analysis consisted of participants who had complete data on both UUI and WWI. Initially, 29,400 individuals were included in the study, and after excluding individuals under 18 years old (n = 11,439) and those with missing data related to pregnancy (n = 190), UUI (n = 3040), and WWI (n = 613), our final analysis comprised 14,118 eligible participants (as shown in Fig. 1).

### Assessment of weight-adjusted-waist index

WWI is a method of measuring obesity that utilizes waist circumference and weight. Higher WWI scores indicate a higher level of obesity. Weight and WC measurements were taken in a mobile examination center (MEC) by certified health technicians. The WWI of every participant was determined by taking the square root of their waist circumference (cm) and dividing it by their weight (kg). Participants were grouped for subsequent analyses based on their WWI quartiles, and WWI was also considered a continuous variable. WWI was regarded as the main factor of exposure in our research. In addition, we employed alternative measures of obesity such as waist circumference (WC) and body mass index (BMI).

### Assessment of urge urinary incontinence

UUI was determined by the response to the question, “During the past 12 months, have you leaked or lost control of even a small amount of urine with an urge or pressure to urinate and you could not get to the toilet fast enough?” UUI severity was characterized by the response to the question, “How frequently does this occur?” At least weekly UUI and at least daily UUI were characterized as variables separate from any overall UUI.

### Assessment of covariates of interest

In our study, we considered potential covariates that could impact the relationship between WWI and UUI. These covariates encompassed gender, age, race, education level, poverty-income ratio (PIR), body mass index (BMI, kg/m^2^), diabetes, and waist circumference (cm). BMI is classified into three categories: < 25, 25–29.9, and ≥ 30 kg/m^2^, corresponding to normal weight, overweight, and obese categories. Smoking status was determined based on a positive response to either of the following questions: "Have you smoked at least 100 cigarettes in your entire life?" or "Do you now smoke cigarettes?" Alcohol use was defined as having consumed a minimum of 12 standard alcoholic drinks within a year^14^. Finally, health insurance coverage was assessed using the question: "Are you covered by health insurance or some other kind of health care plan?".

### Statistical analysis

The guidelines provided by the Centers for Disease Control and Prevention (CDC) were used for all statistical analyses in this study and incorporated NHANES sampling weights to address the complexities of multistage cluster surveys. To evaluate disparities among participants grouped by WWI quartiles, descriptive analyses were conducted using weighted Student's t-tests for continuous variables and weighted chi-squared tests for categorical variables. Means and standard errors (SE) were used to report continuous variables, whereas proportions were presented for categorical variables. To investigate the relationship between WWI and UUI, three distinct models were analyzed using weighted multivariable regression. Model 1 did not involve any modifications for covariates, while Model 2 incorporated adjustments for age, gender, and race. Model 3 was further adjusted for age, gender, race, education level, poverty-income ratio, health insurance coverage, alcohol use, diabetes, and smoking.

To confirm the validity of our findings, we carried out a sensitivity analysis by dividing WWI into four equal groups. To address potential nonlinearity in the data, we used smooth curve fitting and a generalized additive model (GAM). When a nonlinear relationship was observed, we employed a segmented regression model, also known as a two-piecewise linear regression model, to fit each segment and determine the threshold effect. We evaluated the existence of a threshold by conducting a log-likelihood ratio test, which compared a single-line model (without segmentation) with a two-segmented linear regression model. To identify the breakpoint (K)^15^, we employed a two-step recursive approach.

We conducted subgroup analyses to examine the associations between WWI and UUI, stratifying participants by age (< 50/≥ 50 years), gender (male/female), diabetes (yes/no), and BMI (normal weight/overweight/obesity). These stratified variables were regarded as possible effect modifiers that had already been predetermined. To evaluate the diversity of connections among the subgroups, we incorporated an interaction term. Additionally, we employed smooth curve fitting to identify potential nonlinearity in the relationships between WC, BMI, and UUI. When nonlinearity was detected, we applied a two-piecewise linear regression model to explore the presence of threshold effects. In order to handle missing values, the mode and median of the cases that already had missing data for categorical variables and continuous variables, respectively, were imputed. Both R (version 4.2.0) and EmpowerStats (version 4.1) were used for all statistical analyses and statistical significance with a two-sided *P* value < 0.05 was defined.

## Results

### Baseline characteristics

A total of 14,118 individuals were involved in the study, with an average age of 48.05 ± 17.00 years. Of the participants, 49.44% were male, and 50.56% were female. WWI quartiles were defined as follows: Q1 (8.46–10.54), Q2 (10.54–11.12), Q3 (11.12–11.70), and Q4 (11.70–14.79). Among them, 3338 (23.64%) reported any UUI, 838 (5.94%) reported at least weekly SUI, and 313 (2.22%) reported at least daily UUI. And there was a significant increase in overall UUI prevalence with higher WWI quartiles (Q1: 11.15%; Q2: 16.78%; Q3: 23.75%; Q4: 36.91; *P* < 0.0001). Compared to individuals in the bottom quartile of WWI, those in the top quartile exhibited a higher likelihood of being female, older, white, having a lower education level, smoking, consuming excess alcohol, having a lower poverty-income ratio, and having a higher BMI (Table 1).

**Table 1:** Baseline characteristics of study population according to weight-adjusted-waist index tertiles.

Weight-adjusted-waist index	Overall	Q1	Q2	Q3	Q4	*P* value
		(8.46–10.54)	(10.54–11.12)	(11.12–11.70)	(11.70–14.79)	
		N = 3530	N = 3529	N = 3529	N = 3530	
Age(year)	48.05 ± 17.00	37.45 ± 13.64	46.92 ± 15.27	52.59 ± 15.98	58.45 ± 15.79	< 0.0001
Gender, (%)						< 0.0001
Male	49.44	59.60	54.00	47.76	32.39	
Female	50.56	40.40	46.00	52.24	67.61	
Race, (%)						< 0.0001
Mexican American	8.80	5.84	8.81	11.09	10.20	
Other Hispanic	6.13	5.59	6.71	6.73	5.47	
Non-Hispanic White	65.38	64.66	64.05	64.21	69.22	
Non-Hispanic Black	10.88	14.53	9.93	9.35	8.89	
Other Races	8.81	9.38	10.50	8.62	6.21	
Education level, (%)						< 0.0001
Less than high school	12.79	8.70	11.60	14.75	17.48	
High school or GED	23.49	20.26	22.56	24.60	27.64	
Above high school	63.72	71.04	65.84	60.64	54.88	
Smoke, (%)						< 0.0001
Yes	43.65	37.11	42.66	47.62	49.17	
No	56.35	62.89	57.34	52.38	50.83	
Diabetes, (%)						< 0.0001
Yes	11.04	1.88	7.64	13.01	25.44	
No	88.96	98.12	92.36	86.99	74.56	
Alcohol use, (%)						< 0.0001
Yes	69.92	77.55	73.48	68.16	57.38	
No	30.08	22.45	26.52	31.84	42.62	
Health insurance coverage, (%)						< 0.0001
Yes	85.02	82.64	83.67	87.23	87.39	
No	14.98	17.36	16.33	12.77	12.61	
BMI (kg/m^2^)	29.41 ± 7.01	25.07 ± 4.78	28.50 ± 5.35	30.93 ± 6.41	34.58 ± 7.88	< 0.0001
Waist Circumference (cm)	100.42 ± 16.97	86.44 ± 10.82	97.85 ± 11.70	105.63 ± 13.46	116.26 ± 16.30	< 0.0001
Weight (kg)	83.70 ± 22.04	74.94 ± 17.24	82.66 ± 19.48	87.27 ± 22.35	92.59 ± 25.56	< 0.0001
Poverty income ratio	3.03 ± 1.64	3.19 ± 1.66	3.17 ± 1.63	3.02 ± 1.65	2.66 ± 1.58	< 0.0001
UUI overall, (%)	23.64	11.15	16.78	23.75	36.91	< 0.0001
UUI weekly, (%)	5.94	1.09	2.87	4.86	10.72	< 0.0001
UUI daily, (%)	2.22	0.30	0.97	1.85	4.02	< 0.0001

Mean and interquartile range for continuous variables: *P* value was calculated by weighted linear regression model.

% for categorical variables: *P* value was calculated by weighted chi-square test.

GED, general educational development; BMI, body mass index; WWI, weight-adjusted-waist index; UUI, Urge Urinary Incontinence.

### The association between weight-adjusted-waist index and UUI

In Table 2, we present the correlation between WWI and UUI. The results of our study indicated a positive relationship between increased WWI and an increased probability of UUI. This association was consistently observed in both the unadjusted model and the models with minimal or full adjustments. After full adjustment, individuals with a WWI that is one unit higher experienced a 20% rise in the likelihood of UUI (Model 3: OR 1.20, 95% CI 1.13–1.28). Even when WWI was divided into quartiles, this association remained statistically significant. Compared to individuals in the bottom quartile of WWI, those in the top quartile had a significantly higher risk in overall UUI (OR 1.47, 95% CI 1.26–1.72; *P* for trend < 0.0001), weekly UUI (OR 1.87, 95% CI 1.36–2.59; *P* for trend < 0.0001) and daily UUI (OR 1.88, 95% CI 1.05–3.37; *P* for trend = 0.0043) (Table 2).

**Table 2:** Association between weight-adjusted-waist index and UUI.

	Quartile of WWI				*P* for trend	WWI continuous
	Q1	Q2	Q3	Q4		
	(8.46–10.54)	(10.54–11.12)	(11.12–11.70)	(11.70–14.79)		
Overall UUI (OR^a^, 95% CI^b^, P)						
Model 1^c^	Ref	1.67 (1.46, 1.91) < 0.0001	2.59 (2.29, 2.94) < 0.0001	4.46 (3.95, 5.04) < 0.0001	< 0.0001	1.96 (1.87, 2.06) < 0.0001
Model 2^d^	Ref	1.15 (1.00, 1.33) 0.0450	1.36 (1.18, 1.57) < 0.0001	1.75 (1.52, 2.01) < 0.0001	< 0.0001	1.30 (1.22, 1.38) < 0.0001
Model 3^e^	Ref	1.08 (0.93, 1.25) 0.3242	1.23 (1.06, 1.43) 0.0069	1.47 (1.26, 1.72) < 0.0001	< 0.0001	1.20 (1.13, 1.28) < 0.0001
Weekly UUI (OR^a^, 95% CI^b^, P)						
Model 1^c^	Ref	2.05 (1.50, 2.81) < 0.0001	3.95 (2.95, 5.28) < 0.0001	8.33 (6.32, 10.98) < 0.0001	< 0.0001	2.43 (2.23, 2.65) < 0.0001
Model 2^d^	Ref	1.27 (0.92, 1.76) 0.1432	1.74 (1.28, 2.37) 0.0004	2.62 (1.94, 3.53) < 0.0001	< 0.0001	1.53 (1.38, 1.69) < 0.0001
Model 3^e^	Ref	1.10(0.79, 1.55) 0.5666	1.42 (1.02, 1.96) 0.0352	1.87 (1.36, 2.59) 0.0001	< 0.0001	1.32 (1.18, 1.48) < 0.0001
Daily UUI (OR^a^, 95% CI^b^, P)						
Model 1^c^	Ref	2.52 (1.41, 4.50) 0.0019	4.96 (2.89, 8.52) < 0.0001	11.73 (7.02, 19.61) < 0.0001	< 0.0001	2.61 (2.28, 2.99) < 0.0001
Model 2^d^	Ref	1.40 (0.77, 2.52) 0.2680	1.83 (1.04, 3.19) 0.0345	2.85 (1.65, 4.91) 0.0002	< 0.0001	1.52 (1.29, 1.78) < 0.0001
Model 3^e^	Ref	1.15 (0.62, 2.14) 0.6609	1.54 (0.86, 2.77) 0.1459	1.88 (1.05, 3.37) 0.0329	0.0043	1.27 (1.06, 1.53) 0.0091

UUI, urge urinary incontinence.

Insensitivity analysis, the weight-adjusted-waist index was converted from a continuous variable to a categorical variable (quartiles).

^a^OR: Odds ratio.

^b^95% CI: 95% confidence interval.

^c^Model 1: No covariates were adjusted.

^d^Model 2: Adjusted for gender, age, and race.

^e^Model 3: Adjusted for gender, age, race, education level, poverty income ratio, health insurance coverage, alcohol use, diabetes, and smoke.

**Figure 2:**
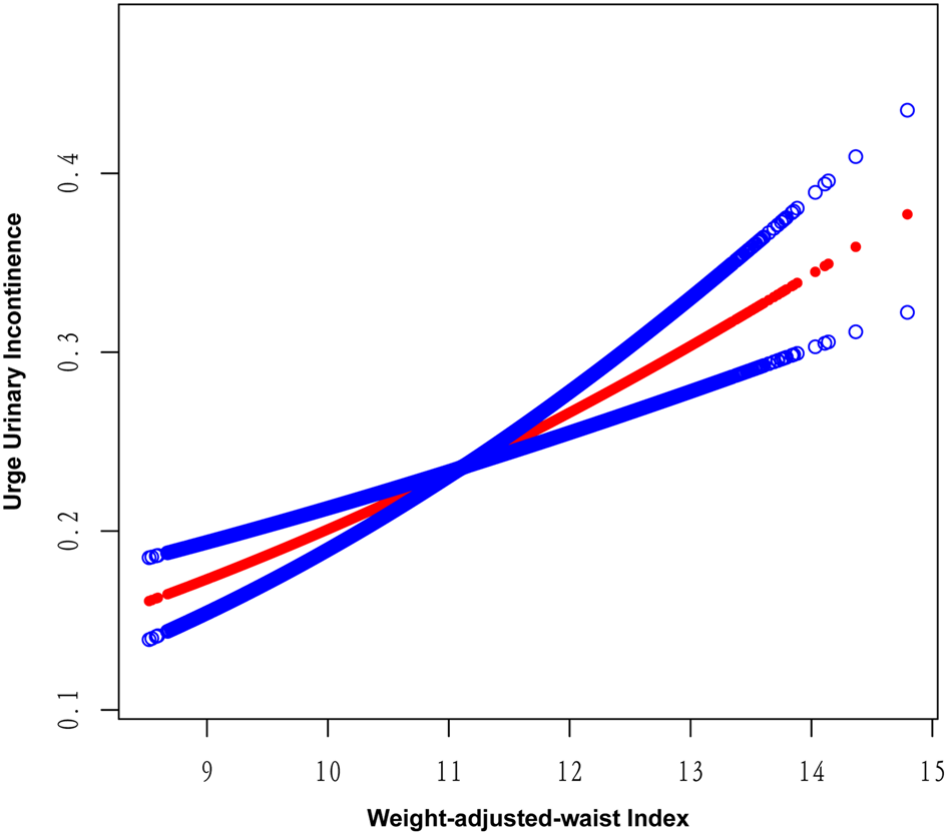
Smooth curve fitting for WWI and UUI.

Age, gender, race, diabetes, smoking status, and poverty-income ratio continued to show a significant association with the likelihood of UUI in the fully adjusted model. Female participants and non-smokers had a 145% and 25% higher risk of UUI, respectively, compared to male participants and smokers. Non-Hispanic White, other Hispanic, and other race groups had a 25%, 10%, and 39% lower likelihood of UUI, respectively, compared to Mexican Americans. The odds of UUI were 30% lower in the non-diabetic population compared to those with diabetes (*P* < 0.0001). With each one-unit increase in age, the odds of UUI increased by 4%, while a one-unit increase in the poverty-income ratio lowered the odds of UUI by 6% (Table 3). Additionally, the findings from the smooth curve fitting analysis suggested that there was no nonlinear correlation between WWI and the likelihood of UUI in the entire sample (Fig. 2).

**Table 3:** Multivariate logistic regression models of UUI.

Variables	OR (95% CI)	*P* value
Weight-adjusted-waist index	1.20 (1.13, 1.28)	< 0.0001
Age(year)	1.04 (1.04, 1.04)	< 0.0001
Female (vs. male)	2.45 (2.21, 2.71)	< 0.0001
Race (vs. Mexican American)		
Other Hispanic	0.75 (0.62, 0.90)	0.0026
Non-Hispanic White	0.90 (0.77, 1.04)	0.1450
Non-Hispanic Black	1.33 (1.14, 1.56)	0.0004
Other races	0.61 (0.50, 0.74)	< 0.0001
Education level (vs. less than high school)		
High school or GED	1.05 (0.92, 1.21)	0.4648
Above high school	1.08 (0.94, 1.23)	0.2618
Smoke (no vs. yes)	1.25 (1.13, 1.38)	< 0.0001
Diabetes (no vs. yes)	0.70 (0.62, 0.79)	< 0.0001
Alcohol use (no vs. yes)	0.95 (0.86, 1.05)	0.2832
Health insurance coverage (no vs. yes)	1.04 (0.91, 1.20)	0.5281
Poverty income ratio	0.94 (0.91, 0.98)	0.0005

The unit for continuous variables and the reference group for categorical variables are provided next to the variables. The OR of UUI was each unit increase of continuous variables and compared with the reference group for categorical variables.

### Subgroup analysis

**Figure 3:**
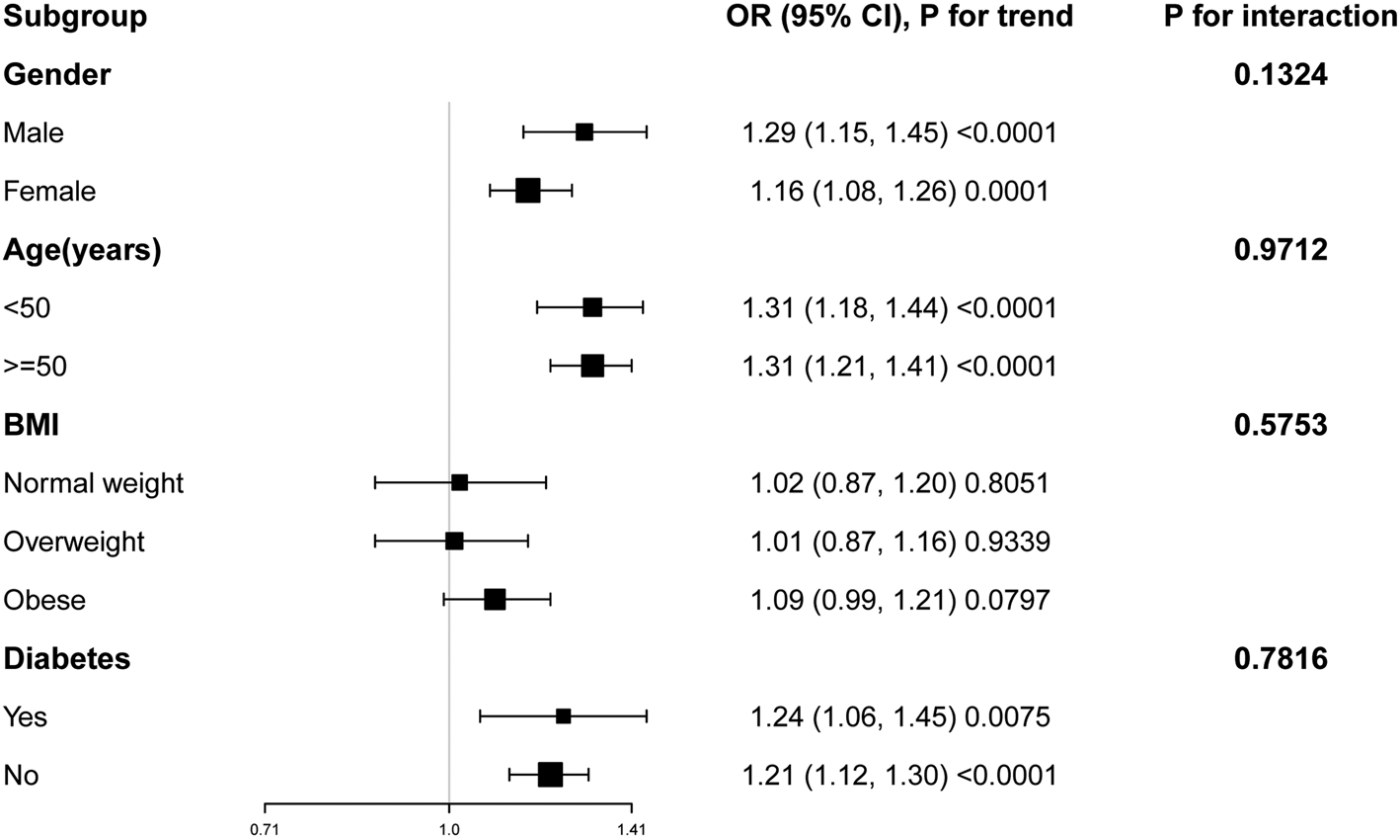
Subgroup analysis for the association between WWI and UUI.

To determine whether the correlation between WWI and UUI held true for other population categories, subgroup analysis was carried out. Our findings indicated that the connection between WWI and UUI was not dependent on specific population characteristics. Figure 3 shows that the positive correlation between WWI and UUI was not substantially affected by any of the stratifications, such as age, gender, BMI, or diabetes status (all *P* values for interaction < 0.05). The positive association remained robust across various subgroups. For instance, we observed a 24% increased likelihood of UUI for each unit increase in WWI in the diabetes subgroup, and this correlation also stayed significant in the non-diabetic subgroup (OR 1.21, 95% CI 1.12–1.30).

### Weight-adjusted-waist index showed a stronger correlation than WC and body mass index for UUI

**Figure 4:**
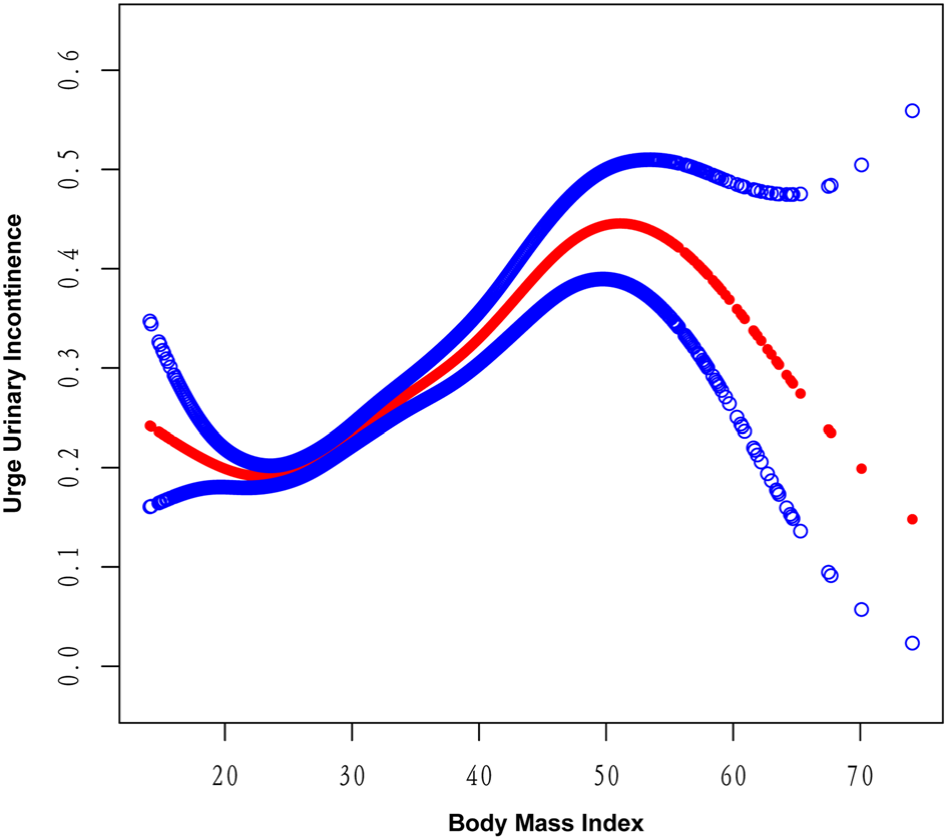
Smooth curve fitting for WWI and BMI.

**Figure 5:**
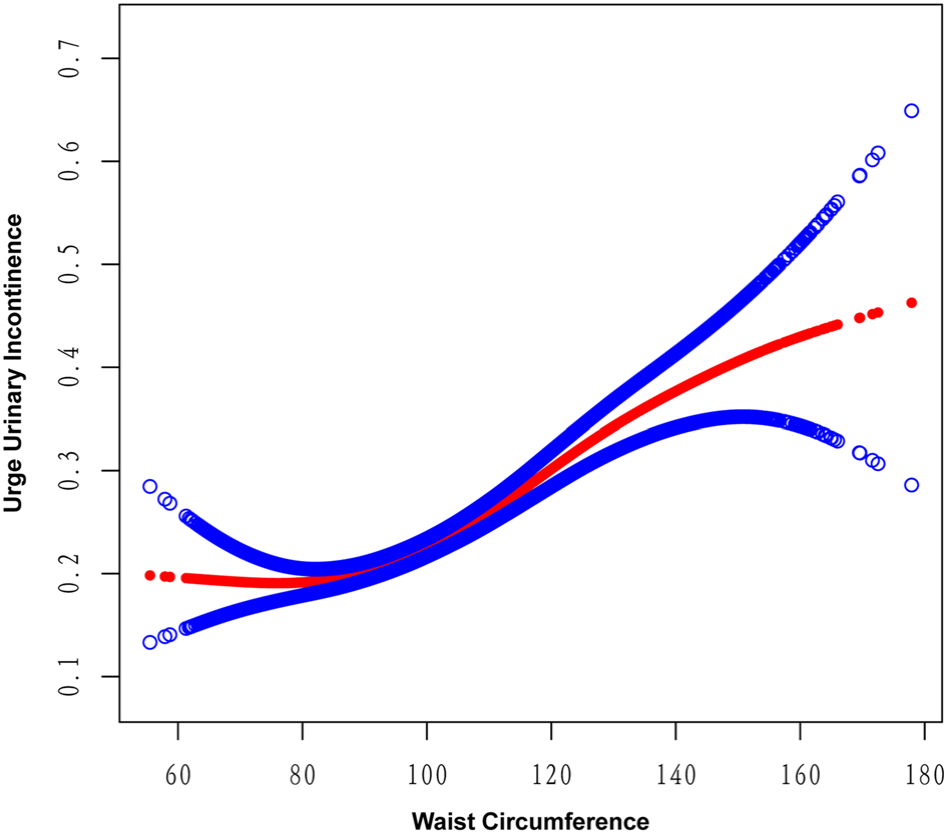
Smooth curve fitting for WWI and WC.

The smooth curve fitting technique uncovered a lack of linearity in the relationship between BMI and UUI (Fig. 4) as well as between WC and UUI (Fig. 5). Subsequently, we employed the segmented regression model to identify the threshold effect (Table 4). A breakpoint of 22.8 kg/m^2^ was identified in the association between BMI and UUI. A negative correlation was observed below this threshold (OR 0.95, 95% CI 0.90–1.00), whereas a positive correlation was noted above this threshold (OR 1.04, 95% CI 1.04–1.05). Similarly, the breakpoint for WC and UUI was 79 cm. On the left side of this breakpoint, WC exhibited a negative association with the likelihood of UUI (OR 0.96, 95% CI 0.93–0.99), whereas on the right side, the association was positive (OR 1.02, 95% CI 1.01–1.02). When conducting the nonlinear model, we also found consistent positive association between WWI and UUI (Table 4). The correlation between WWI and UUI risk was significantly stronger compared to BMI and WC values to the right of the respective breakpoints (WWI: OR 1.20; BMI: OR 1.04; WC: OR 1.02). This suggests that WWI might serve as a more effective predictive marker of UUI risk than other obesity markers like BMI and WC.

**Table 4:** Threshold effect analysis of WWI, BMI and WC on UUI using a two-piecewise linear regression model.

	WWI	BMI	WC
Fitting by standard linear model			
OR^a^ (95% CI^b^)	1.20 (1.13, 1.28)	1.04 (1.03, 1.05)	1.02 (1.01, 1.02)
* P* value	< 0.0001	< 0.0001	< 0.0001
Fitting by two-piecewise linear model			
Breakpoint (K)	12.11	22.8	79
OR1(< K)	1.23 (1.13, 1.33) < 0.0001	0.95 (0.90, 1.00) 0.0537	0.96 (0.93, 0.99) 0.0190
OR2(> K)	1.10 (0.88, 1.37) 0.3992	1.04 (1.04, 1.05) < 0.0001	1.02 (1.01, 1.02) < 0.0001
OR2/OR1	0.90 (0.69, 1.16) 0.4146	1.10 (1.04, 1.17) 0.0008	1.06 (1.02, 1.09) 0.0008
Logarithmic likelihood ratio test * P* value	0.414	< 0.001	0.001

WWI, weight adjusted waist index; BMI, body mass index; WC, waist circumference; UUI, urge urinary incontinence. Age, gender, race, education level, poverty income ratio, health insurance coverage, alcohol use, diabetes, and smoke were adjusted.

^a^OR: Odds ratio.

^b^95% CI: 95% confidence interval.

## Discussion

The main objective of this research was to investigate the relationship between the Waist-to-Weight Index (WWI) and Urge Urinary Incontinence (UUI) among residents in the United States. In this study with 14,118 participants, it was observed that individuals with higher WWI were more likely to experience UUI. Subgroup analyses and interaction tests revealed that this association held true across different population groups. Notably, the relationship between WWI and UUI proved stronger than the associations between UUI and other common obesity indicators like BMI and waist circumference (WC), indicating that WWI may be a more reliable predictor of UUI than other obesity markers. Our results suggest that WWI could serve as a valuable predictor of UUI incidence, and managing obesity as assessed by WWI may help reduce the risk of UUI. These findings suggest that WWI may serve as a valuable predictive factor for the onset of UUI, and the management of obesity, as indicated by WWI assessment, could potentially contribute to reducing the risk of UUI.

A growing body of evidence strongly supports the pivotal role of obesity in the pathogenesis of UUI. Previous studies have consistently reported that a BMI of ≥ 30 is associated with UI in both men and women^6^. Long-term follow-up studies over five to ten years have shown that for every 1 kg/m^2^ increase in body mass index (BMI), the odds of developing UUI increase by approximately 3% to 14%^7^. Women with larger waist circumferences are more likely to experience lower urinary tract symptoms (LUTS), including urinary incontinence^8^. In a large-scale, population-based, 4-year longitudinal study in China, it was found that women with higher baseline BMIs had a lower rate of UUI remission four years later^9^. Therefore, accurate assessment of obesity is of paramount importance in reducing the risk of UUI.

There are multiple potential explanations for this phenomenon. One possible explanation is that obesity is often linked to a variety of diet-related chronic diseases, such as diabetes, hypertension, and cardiovascular conditions^16–18^. Since individuals with UUI typically present with more medical comorbidities compared to those without UUI, suggesting that overall health status may influence the occurrence of UUI symptoms. This relationship highlights the importance of obesity in the mechanisms behind the promotion of urinary incontinence symptoms, as well as the need to consider an individual's overall health status in an integrated manner when recognizing and treating UUI. It also emphasizes that adopting a comprehensive health management strategy to reduce obesity and prevent associated chronic diseases may help to reduce the risk of urinary incontinence and the severity of symptoms.

Beyond the link between diet-related chronic diseases and obesity, obesity may directly exacerbate UUI symptoms by causing stretching and weakening of the pelvic floor muscles^19^. This phenomenon involves several physiological and anatomical factors. First, obesity causes weight gain, which imposes an additional burden on the pelvic floor muscles. Over time, this additional burden may lead to hyperextension of the pelvic floor muscles, causing them to weaken and lose their function of supporting urinary control. Weakened pelvic floor muscles may lead to relaxation of the urethral sphincter, which increases the risk of inadvertent urinary leakage. In addition, obesity may lead to the accumulation of abdominal fat, which increases intra-abdominal pressure. This additional intra-abdominal pressure may exacerbate the relaxation of the urethral sphincter, making it more difficult to effectively control the discharge of urine. Intra-abdominal pressure may also have a direct effect on bladder capacity and perception, leading to urine retention and urgency. All of these physiologic changes can directly contribute to the exacerbation of UUI symptoms.

Stress could play another intermediary role in this association. Individuals with obesity may experience weight discrimination^20^, which is highly prevalent and can induce stress^21,22^. Stress is believed to have a role in the pathophysiology of Overactive Bladder (OAB) and UUI because hypothalamic-pituitary axis function differs markedly in people with OAB^23^. Preclinical models have demonstrated worsened OAB symptoms following cortisol administration^24^. Furthermore, prior research has found that depression can predict the occurrence of urinary incontinence, possibly due to elevated levels of circulating cortisol and catecholamines, which could serve as potential physiological factors in bladder changes^25,26^. Consequently, the association between obesity and UUI appears to be multifactorial and bidirectional in nature.

Various indicators have been utilized to assess obesity, with a particular focus on the detrimental abdominal visceral fat mass. While BMI is extensively utilized to evaluate body composition, it falls short in distinguishing between lean body mass and fat mass. An alternative method to evaluate increased abdominal fat is waist circumference (WC), however, it is unable to assess increased abdominal fat, but it, too, cannot differentiate between visceral and subcutaneous fat. WWI, a newly developed obesity metric, is proposed as a more accurate predictor of total body fat percentage compared to traditional BMI-based equations. WWI has been widely applied in multiple areas^12,27^. WWI is shown to be the most strongly associated indicator with the risk of cardiovascular and metabolic diseases, as well as mortality, surpassing BMI, WC, WHtR, and ABSI in a large-scale cohort study involving 465,629 participants^28^. Based on Wen et al.'s research, it was found that female infertility had a stronger correlation with WWI compared to other indicators of obesity like WC, BMI, and ABSI^29^. The research reveals a non-linear positive association between WWI and urinary incontinence (UUI), and sensitivity analysis based on WWI quartiles further highlights the dose–response relationship between WWI and UUI. Most notably, the link between WWI and the likelihood of UUI is significantly stronger than that of other obesity indicators like BMI and WC (WWI: OR 1.20; BMI: OR 1.04; WC: OR 1.02). In summary, this study lends support to the idea that WWI may serve as a valuable predictive indicator for obesity-related diseases. In addition, although Pang^30^ found the relationship between visceral adipose index and stress urinary incontinence (SUI), there is no research to study the relationship between WWI and SUI and further research is required.

This study has numerous benefits. The research is based on nationwide data and incorporates sample weighting, making the study's findings widely applicable to the overall U.S. population. The results were confirmed to be robust through subgroup analyses, thanks to the inclusion of covariate adjustments in the regression analysis and the utilization of a large sample size. Finally, the study further reveals that the waist-to-weight ratio is more strongly correlated with urinary incontinence (UUI) than the body mass index (BMI). However, several limitations also need to be declared. Despite adjusting for multiple confounding factors, it's impossible to entirely eliminate the influence of other confounding variables. Thus, caution is warranted when interpreting the study's results. In addition, WC which is not routinely measured in clinic and therefore limits clinical utility of WWI. Most importantly, causality cannot be determined because of the cross-sectional study design.

## Conclusions

This investigation has uncovered a connection between elevated WWI levels and an increased risk of UUI. Notably, there is a stronger correlation between WWI and UUI compared to other obesity indicators and UUI. We hypothesize that this has important implications for the prevention and treatment of UUI. Future studies are needed to further test this hypothesis.

## Data Availability

The datasets used and/or analysed during the current study available from the corresponding author on reasonable request.
